# Natural variation of *RGN1a* regulates grain number per panicle in *japonica* rice

**DOI:** 10.3389/fpls.2022.1097622

**Published:** 2022-12-13

**Authors:** Quan Zhang, Jianyin Xie, Xueqiang Wang, Miaosong Liu, Xiaoyang Zhu, Tao Yang, Najeeb Ullah Khan, Chen Sun, Jinjie Li, Zhanying Zhang, Zichao Li, Hongliang Zhang

**Affiliations:** ^1^ State Key Laboratory of Agrobiotechnology/Beijing Key Laboratory of Crop Genetic Improvement, China Agricultural University, Beijing, China; ^2^ Sanya Institute of China Agricultural University, Sanya Nanfan Research Institute of Hainan University, Sanya, China

**Keywords:** GWAS, grain number per panicle, breeding, haplotype, rice

## Abstract

The grain number per panicle (GNP) is an important yield component. Identifying naturally favorable variations in GNP will benefit high-yield rice breeding. Here, we performed a genome-wide association study using a mini-core collection of 266 cultivated rice accessions with deep sequencing data and investigated the phenotype for three years. Three genes, i.e., *TOTOU1* (*TUT1*), *Grain height date 7* (*Ghd7*), and *Days to heading 7*/*Grain height date 7.1*/*Pseudo-Response Regulator37* (*DTH7/Ghd7.1*/*OsPRR37*), which regulate GNP, were found in the quantitative trait loci (QTL) identified in this study. A stable QTL, *qGNP1.3*, which showed a strong correlation with variations in GNP, was repeatedly detected. After functional and transgenic phenotype analysis, we identified a novel gene, *regulator of grain number 1a* (*RGN1a*), which codes for protein kinase, controlling GNP in rice. The *RGN1a* mutation caused 37.2%, 27.8%, 51.2%, and 25.5% decreases in grain number, primary branch number per panicle, secondary branch number per panicle, and panicle length, respectively. Furthermore, breeding utilization analysis revealed that the additive effects of the dominant allelic variants of *RGN1a* and *DTH7* played a significant role in increasing the grain number per panicle in *japonica* rice. Our findings enrich the gene pool and provide an effective strategy for the genetic improvement of grain numbers.

## Introduction

Grain yield in rice (*Oryza sativa* L.) comprises three components: grain number per panicle (GNP), grain weight, and effective panicle number. Increasing the number of grains per panicle is an effective strategy for improving rice yield in modern breeding. In recent years, many genes that affect panicle development have been identified. However, rice germplasm resources still contain many potentially excellent alleles for controlling the number of grains to be identified. Therefore, enriching the gene pool is important for yield improvement in cultivated rice.

Over the decades of rice research, a series of important grain-number genes have been cloned from germplasm resources. *Grain number 1a* (*Gn1a*) was identified in the near-isogenic lines of Habataki in the Koshihikari background, regulating grain numbers by influencing cytokinin accumulation in inflorescence meristems (Ashikari et al., 2005). *IDEAL PLANT ARCHITECTURE 1*/*SQUAMOSA PROMOTER BINDING PROTEIN-LIKE 14* (*IPA1/OsSPL14*) was detected in two pairs of rice combinations (Taichung Native 1 (TN1) and Shaoniejing (SNJ), Nipponbare (NIP) and ST-12), affecting the tiller number and grain number per panicle (Jiao et al., 2010; Miura et al., 2010). *FRIZZY PANICLE* (*FZP*)/*SMALL GRAIN AND DENSE PANICLE 7* (*SGDP7*)/*CONTROL OF SECONDARY BRANCH 1* (*COS1*) controls secondary branches per panicle, thus influencing grain number. The functional variation site located in the upstream regulatory region of *FZP* has been identified using map-based cloning (Bai et al., 2017; Huang et al., 2018). *NUMBER OF GRAINS 1* (*NOG1*) was isolated from wild rice, and upregulation *of NOG1* expression significantly increased the number of grains per panicle (Huo et al., 2017).

In addition to the previously mentioned genes, map-based cloning in mutants identified many genes associated with grain number. The mutation of *LAX PANICLE1* (*LAX1*) resulted in the lateral spikelets being abolished (Komatsu et al., 2003). *LAX PANICLE2* (*LAX2*)/*GRAIN NUMPER PER-PANICLE 4* (*GNP4*) was also cloned from loss-of-function mutants and regulated reproductive branching (Tabuchi et al., 2011; Zhang et al., 2011a). *DROUGHT AND SALT TOLERANCE* (*DST*) in the gain-of-function mutant *reg1* increased panicle branches and grain numbers (Li et al., 2013). *O. sativa SHORT INTERNODES1* (*OsSHI1*) was isolated from the *shi1* mutant (^60^Co-γ irradiation-induced in 93-11 background), modulating IPA1 transcriptional activity to influence plant architecture and grain number per panicle (Duan et al., 2019). Both *GRAIN SIZE AND NUMBER1* (*GSN1*) and *ERECTA1* (*OsER1*) act upstream of the OsMKKK10-OsMKK4-OsMPK6 cascade while exhibiting the opposite grain number regulation pattern (Guo et al., 2018; Guo et al., 2020a). The *REGULATOR OF GRAIN NUMBER1* (*RGN1*) was obtained from a rare rice germplasm with abnormal panicle branches, affecting grain number and panicle architecture (Li et al., 2022).

With the development of sequencing technology, genes controlling grain number have been discovered through the MutMAP approach or genome-wide association study (GWAS) relying on high-throughput sequencing data. *LARGE1* and *LARGE2* were isolated from the F_2_ population by MutMAP analysis. *LARGE1* encodes the Mei2-like protein, and its overexpression lines positively regulate grain number per panicle and reduce grain size and weight (Lyu et al., 2020). Mutation of *LARGE2*, encoded by a HECT-domain E3 ubiquitin ligase, results in a large panicle and an increase in grain number (Huang et al., 2021). Given the complexity of the grain number per panicle, a few genes were mapped using GWAS. Only *Gnd5*, a novel GRAS transcription factor that positively regulates grain number per panicle, has been identified using GWAS of the *japonica* population (Cui et al., 2022).

In this study, we identified three genes, *TOTOU1* (*TUT1*), *Grain height date 7* (*Ghd7*), and *Days to heading 7*/*Grain height date 7.1*/*Pseudo-Response Regulator37* (*DTH7/Ghd7.1*/*OsPRR37*), through a genome-wide association study, which have been reported to regulate grain number. Meanwhile, *qGNP1.3*, a stable signal segment in the whole genome Manhattan map detected multiple times over years of studying the phenotype, and *regulator of grain number 1a* (*RGN1a*) has been shown to participate in panicle development. Breeding utilization analysis showed that aggregating the favorable alleles of *RGN1a* and *DTH7* further improved grain number in the *japonica* population.

## Materials and methods

### Plant materials and growth conditions

A panel of 266 *Oryza sativa* accessions was used from the core collection (Zhang et al., 2011b). All accessions were planted in Sanya, Hainan Province (18°20′N), under normal cultivation conditions in 2010, 2012, and 2013. Each variety (30 plants) was grown in three rows of 10 plants per row. The main panicles of five plants per variety in the middle row were randomly selected to determine panicle phenotype statistics. The average GNP of five plants was used for the analysis. The three years of phenotypic data were marked as 2010_HN, 2012_HN, and 2013_HN.

### Genome sequencing

The sequencing data of the 266 *Oryza sativa* accessions were obtained from the 3,000 Rice Genome Project (3K-RG) database (Wang et al., 2018), which had an average sequencing depth of 14x and generated > 10 million single nucleotide polymorphisms (SNPs) when compared with the Nipponbare reference genome.

### Population genetic analysis

A total of 4,625,141, 3,562,186, and 3,149,160 high-quality SNPs with missing rates ≤ 50% and minor allele frequencies ≥ 2% were first identified in the full, *indica* and *japonica* populations. Principal component (PC) and kinship analyses were conducted using GAPIT to verify the population structure (Tang et al., 2016). A total of 514,177 SNPs (missing rates ≤ 50% and minor allele frequencies ≥ 5%) were filtered using linkage disequilibrium (LD) pruning and used to construct the neighbor-joining tree in MEGA 7.0 (Kumar et al., 2016).

### GWAS

GWAS was performed using a compressed mixed linear model (CMLM) with the first three PCs in GAPIT software (Tang et al., 2016). The conditional permutation test was executed as previously reported to define the suggestive thresholds (Zhao et al., 2018), and 196,787, 228,287, and 123,723 effective numbers of independent SNPs were first calculated and obtained by PLINK 1.9 (–indep-pairwise: 50 5 0.3, window size 50 SNPs, step size 5 SNPs, r^2^ ≥ 0.3) in the full, *indica*, and *japonica* populations, respectively (Purcell et al., 2007; Guo et al., 2020b). Then, the formula “-log_10_ (1/effective number of independent SNPs)”, as previously described, was used to set a significant threshold. Combining the above two methods, we set a significance threshold of *P* = 10^-5^ at a genome-wide level. Quantitative trait loci (QTL) detection using this method identified a region containing at least three clustered significant SNPs within a distance of < 170 kb from one another (Huang et al., 2010; Zhao et al., 2018). Based on genome structure annotation information from MSU-RGAP 7.0, non-synonymous SNPs were annotated using SnpEff (Cingolani et al., 2012). They were then separated from all SNPs identified in the 266 accessions using an in-house Perl script. LD heatmaps of target regions in the GWAS were constructed using “LD heatmap” in the R package (Shin et al., 2006).

### Haplotype analysis

Given that *TUT1*, *Ghd7*, and *DTH7* all existed functional SNPs (-log(*P*) ≥ 3) in this study, haplotype analysis was based on the SNPs (*P* ≤ 10^-3^) in the 2 k gene promoter and exons. Grain number per panicle was evaluated after harvest. Haplotypes, which were used for statistical testing, contain four varieties at least.

### Protein sequence analysis of RGN1a and RGN1b

Homologous protein sequences of RGN1a and RGN1b were downloaded from NCBI (National Center for Biotechnology Information, https://www.ncbi.nlm.nih.gov/). Amino acid sequence alignments were assessed with DNAMAN version 6.0 software (Lynnon Corporation, San Ramon, CA, USA). A neighbor-joining tree with sequence homology to RGN1a and RGN1b was constructed in MEGA 7.0.

### Analysis of T-DNA insertion mutants

After the candidate gene analysis of grain number per panicle, we obtained the T-DNA insertion mutant from the POSTECH Biotech Center, Republic of Korea. The plants were sown at the Shangzhuang Experimental Farm of China Agricultural University in Beijing. We designed specific primers in the gene body (LP and RP) and T-DNA specific primers (RB) to identify the genotype of mutants.

### qRT-PCR

Total RNA was extracted from young panicle. qRT-PCR was performed using TB Premix Ex Taq II with ROX Reference Dye II (Takara, #RR820A) on the Applied Biosystems 7500 Fast Real-Time PCR System. Relative gene expression level was analyzed using the comparative critical threshold (△△Ct) method (Livak and Schmittgen, 2001).

### Primers

Primers used in the study are listed in **Supplementary Table 1**.

### Accession numbers

The genes used in this study can be found in the Rice Genome Annotation Project (http://rice.uga.edu/home_overview.shtml) with the following accession numbers: *RGN1a* (*LOC_Os01g49580*), *RGN1b* (*LOC_Os01g49614*), *TUT1* (*LOC_Os01g11040*), *Ghd7* (*LOC_Os07g15770*), and *DTH7*/*Ghd7.1*/*OsPRR37* (*LOC_Os07g49460*).

## Results

### Population structure and phenotypic variation of 266 accessions for grain number per panicle

**Figure 1:**
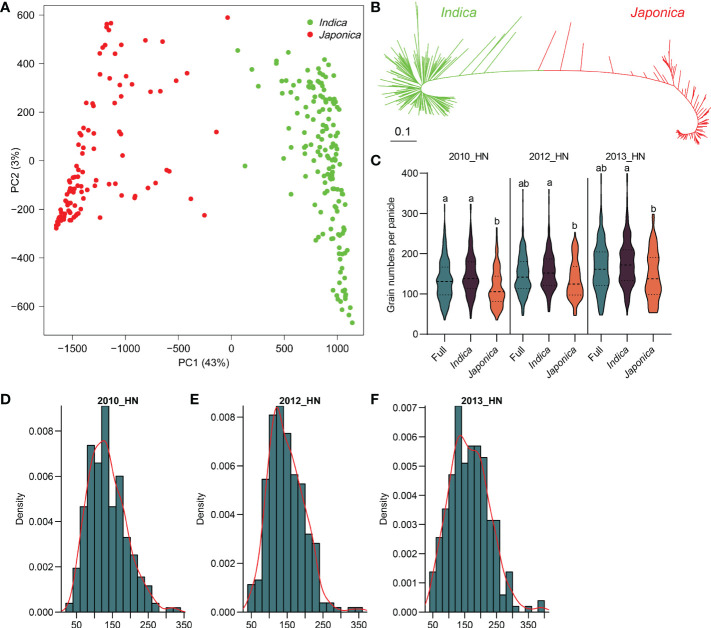
Population structure of 266 rice accessions. **(A)** Principal component analysis (PCA) of different subpopulations. **(B)** Neighbor-joining tree for all accessions; green lines represent *indica*, and red lines represent *japonica* rice. **(C)** The phenotype statistics of grain number per panicle in full, *indica*, and *japonica* populations over three years. The numbers above violins are mean phenotypic values, and different letters indicate significant differences at *P* < 0.05 according to one-way ANOVA. **(D–F)** The distribution of the grain number per panicle in full population among different years.

We retrieved 4,625,141 high-quality SNPs (missing rates ≤ 50% and minor allele frequencies ≥ 2%) from the 3K-RG project as genotypes. Principal component and kinship analyses using 4,625,141 SNPs revealed an apparent population structure. The *indica* and *japonica* subpopulations were clearly separated (**Figure 1A**; **Supplementary Figure 1**). The neighbor-joining tree using 514,177 SNPs also showed distinct differentiation between the two subspecies (**Figure 1B**).

As the population structure analysis showed two *O. sativa* subspecies, abundant differences in GNP existed between the *indica* and *japonica* subspecies. Overall, *indica* rice had more grains per panicle than *japonica* rice did (**Figure 1C**). The variation of GNP in the *indica* variety ranged from 38.9 to 323.4 (2010), 55.8 to 359.7 (2012), and 47.5 to 399.1 (2013), and from 35.2 to 265.1 (2010), 46.7 to 253.7 (2012), and 53.2 to 298.5 (2013) in *japonica*. The phenotypic data were normally distributed and suitable for association analysis (**Figures 1D–F**; **Supplementary Figure 2**).

### Identifying the QTLs related to the grain number per panicle in rice

**Figure 2:**
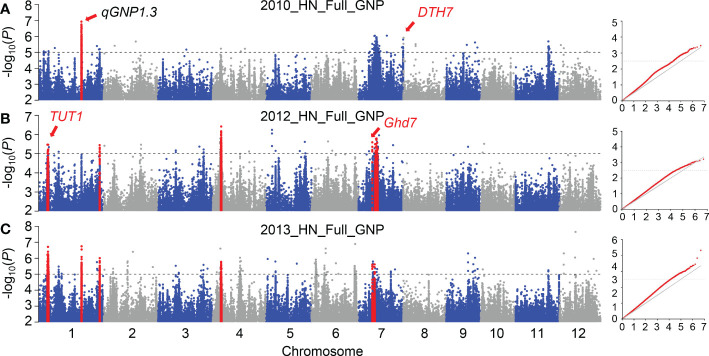
GWAS results of GNP in different years. Quantile–quantile plots and Manhattan plots for the GWAS in 2010 **(A)**, 2012 **(B)**, and 2013 **(C)** using CMLM. Red points in the Manhattan plot represent the QTLs that were identified at least two years. In quantile-quantile plots, red points show the CMLM model. In the Manhattan plots, the gene in red was previously cloned. A dotted horizontal line for each figure indicates the significance threshold (*P* = 10^−5^).

We used a rigorous compressed mixed linear model (CMLM) for GWAS to identify important QTLs for GNP. Using the phenotype of GNP in 2010, 210, 12, and 325 SNPs were identified by GWAS at -log(*P*) ≥ 5 in the full, *indica*, and *japonica* populations, respectively (**Figure 2A**; **Supplementary Figure 3**); 176, 121, and 10 significant SNPs (*P* ≤ 10^-5^) were identified by GWAS on GNP_2012 in the full, *indica*, and *japonica* populations, respectively (**Figure 2B**; **Supplementary Figure 3**); and 363, 14, and 7 significant SNPs were identified by GWAS on GNP_2013 in the full, *indica*, and *japonica* populations, respectively (**Figure 2C**; **Supplementary Figure 3**). A total of 126 and 29 significant SNPs were detected in the two and three GWAS populations, respectively. In total, 44 SNPs associated with GNP were repeatedly detected from the full population among different years; only 8 and 3 SNPs were detected repeatedly from the *indica* and *japonica* populations, respectively (**Supplementary Figure 4**). Given that the linkage disequilibrium decay value reported in rice is up to 167 kb (Huang et al., 2010), we defined a QTL as having at least three significant SNPs within distances ≤ 170 kb between adjacent ones (Zhao et al., 2018). Phenotypic data from three years were used to detect QTLs successively. In total, 17 QTLs were detected in 2010, including 9 associated regions in the full population, one in the *indica* population, and seven in the *japonica* population; 19 QTLs were detected in 2012, including 9, 8, and 2 associated regions in the full, *indica*, and *japonica* populations, respectively; and 20 QTLs were detected in 2013, including 19 associated regions in the full population, and 1 QTL was detected in the *japonica* population (**Table 1**; **Supplementary Table 2**).

**Table 1:** Description of 27 QTLs for GNP in full population of 2010_HN, 2012_HN, and 2013_HN.

QTL	Env	Chr	Left_Position	Right_Position	Leader_SNP	P-value	Cloned Gene
qGNP1.1	2013_HN_Full	Chr1	5849202	6587028	Chr1_6230515	1.93781E-07	TUT1; ES1
	2012_HN_Full	Chr1	5869453	6376313	Chr1_6370220	6.46E-06	
qGNP1.2	2013_HN_Full	Chr1	13326106	13335641	Chr1_13335641	6.96E-06	
qGNP1.3	2010_HN_Full	Chr1	28396698	28559232	Chr1_28430351	1.20111E-07	
	2013_HN_Full	Chr1	28441313	28561515	Chr1_28527948	1.79188E-07	
qGNP1.4	2012_HN_Full	Chr1	40482831	40566652	Chr1_40482831	3.60159E-06	
	2013_HN_Full	Chr1	40519665	40594290	Chr1_40555127	1.48E-06	
qGNP2.1	2013_HN_Full	Chr2	6133795	6176257	Chr2_6176257	4.45757E-06	
qGNP2.2	2013_HN_Full	Chr2	15590098	15973327	Chr2_15650305	1.93E-06	
qGNP3.1	2012_HN_Full	Chr3	30250873	30336491	Chr3_30314503	6.03018E-06	
qGNP4.1	2012_HN_Full	Chr4	4803513	5048197	Chr4_5048197	1.59354E-06	
qGNP4.2	2012_HN_Full	Chr4	5374900	5707781	Chr4_5583572	3.81691E-07	
	2013_HN_Full	Chr4	5374900	5774031	Chr4_5524548	1.69161E-06	
qGNP4.3	2013_HN_Full	Chr4	18066568	18159709	Chr4_18159709	2.90E-06	
qGNP4.4	2013_HN_Full	Chr4	18615727	18726211	Chr4_18615923	4.55E-06	
qGNP5.1	2012_HN_Full	Chr5	3759829	3774726	Chr5_3774726	5.71725E-07	
qGNP5.2	2013_HN_Full	Chr5	6032070	6190305	Chr5_6032070	1.60872E-06	
qGNP6.1	2013_HN_Full	Chr6	2838329	3321404	Chr6_2964098	1.20725E-06	
qGNP6.2	2013_HN_Full	Chr6	8596196	8632881	Chr6_8632881	4.81E-06	
qGNP6.3	2013_HN_Full	Chr6	28683216	28694267	Chr6_28687115	4.38E-06	
qGNP7.1	2012_HN_Full	Chr7	9139870	9141469	Chr7_9139870	4.40E-06	Ghd7
	2013_HN_Full	Chr7	9139870	9236614	Chr7_9141382	2.56804E-06	
qGNP7.2	2013_HN_Full	Chr7	9482294	9575043	Chr7_9482294	2.23E-06	
qGNP7.3	2010_HN_Full	Chr7	10355885	10539131	Chr7_10355885	5.33E-06	
	2013_HN_Full	Chr7	10589671	10823047	Chr7_10589671	3.60E-06	
	2010_HN_Full	Chr7	10765889	10823321	Chr7_10823321	9.08837E-07	
qGNP7.4	2012_HN_Full	Chr7	11010900	12049168	Chr7_12292816	5.09E-06	
	2010_HN_Full	Chr7	11591862	11687625	Chr7_11591862	2.84E-06	
	2010_HN_Full	Chr7	11873885	12049168	Chr7_11981983	1.12799E-06	
qGNP7.5	2012_HN_Full	Chr7	12292816	12694639	Chr7_12298575	2.47212E-06	
	2010_HN_Full	Chr7	12396688	12916946	Chr7_12396688	2.26019E-06	
qGNP7.6	2010_HN_Full	Chr7	18792445	18792470	Chr7_18792445	9.08609E-07	
qGNP7.7	2013_HN_Full	Chr7	21465423	21468954	Chr7_21468954	7.78E-06	
qGNP7.8	2010_HN_Full	Chr7	29456155	29648694	Chr7_29586936	1.58871E-06	DTH7; Ghd7.1
qGNP9.1	2013_HN_Full	Chr9	14560809	14581570	Chr9_14581570	6.24E-06	
qGNP11.1	2010_HN_Full	Chr11	21691728	21698304	Chr11_21698268	2.08144E-06	
qGNP12.1	2013_HN_Full	Chr12	2860997	2941857	Chr12_2941857	4.94984E-07	

### Cloned rice grain number genes *TUT1*, *Ghd7*, and *DTH7* showed significantly associated signals in the full population

**Figure 3:**
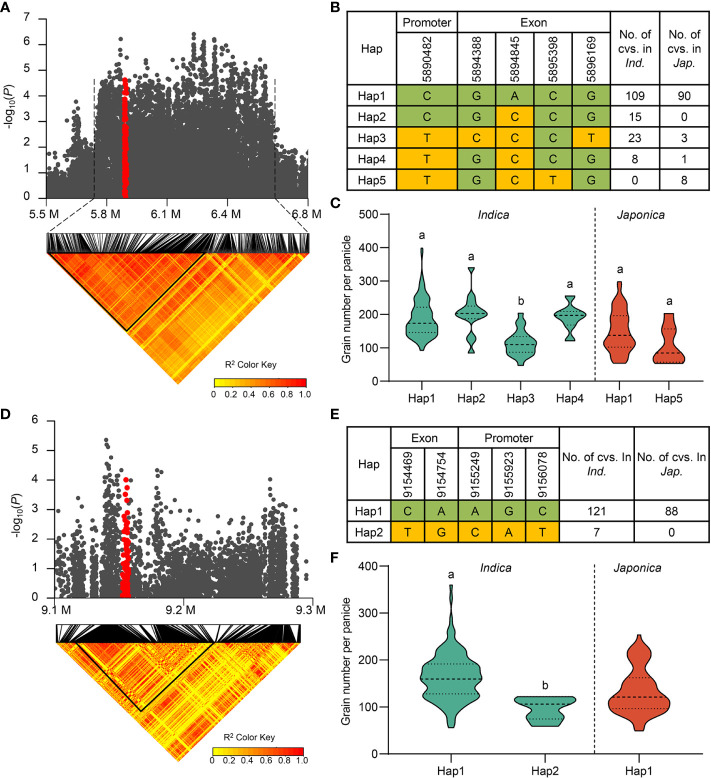
Exploration of *TUT1* and *Ghd7* for grain number per panicle. **(A)** Regional Manhattan plot (top) and pairwise LD analysis (bottom) of *qGNP1.1* containing *TUT1* for GNP. Red dots represent all SNPs within *TUT1*. **(B)** Different haplotypes of *TUT1* in the *indica* and *japonica* subgroups. **(C)** Comparison of GNP traits among haplotypes of *TUT1* in *indica* and *japonica.* **(D)** Regional Manhattan plot (top) and pairwise LD analysis (bottom) of *qGNP7.1* containing *Ghd7* for GNP. Red dots represent all the SNPs within *Ghd7*. **(E)** *Ghd7* haplotypes in the *indica* and *japonica* subgroups. **(F)** Comparison of GNP trait among haplotypes of *Ghd7* in *indica* and *japonica.* The yellow and green in **(B, D)**, respectively, represent major and minor alleles. In **(C, F)**, the green violins represent *indica*, and the red violins represent *japonica* rice, and different letters indicate significant differences (*P* < 0.05) detected by one-way ANOVA.

To verify the reliability of our results, we first checked whether the reported genes were located in candidate QTLs. We found that *TUT1* in *qGNP1.1*, *Ghd7* in *qGNP7.1*, and *DTH7* in *qGNP7.8*, were significantly associated with the grain number per panicle (**Table 1**; **Figures 3A, D**; **Supplementary Figure 5A**).

Mutant *tut1* negatively regulates panicle development in rice and decreases the spikelet number per panicle (Bai et al., 2015). The *TUT1* haplotype in *qGNP1.1* was analyzed using SNPs (*P* ≤ 10^-3^) in exons (non-synonymous SNPs) and the 2 k promoter. There were a total of five haplotypes in the full population (**Figure 3B**). The average GNP values of three favorable haplotypes (*TUT1* ^Hap1^, *TUT1* ^Hap2^, and *TUT1* ^Hap4^) were 65.1%, 76.1%, and 68.3% higher than that of the inferior haplotype *TUT1* ^Hap3^ in the *indica* population, respectively. However, there was no difference in the *japonica* subpopulation (**Figure 3C**).

*Ghd7* encodes a CCT protein, a core factor that regulates heading date and plant height and increases the panicle branch in rice (Xue et al., 2008; Weng et al., 2014). In this study, we found that *Ghd7* is located in *qGNP7.1*. There were three significant SNPs in the promoter region of *Ghd7* and two significant non-synonymous SNPs in the exon. Using the five SNPs, we identified two *Ghd7* haplotypes in 216 rice accessions. *Ghd7* ^Hap2^ only existed in the *indica* population, and *Ghd7* ^Hap1^ exhibited a better grain-number phenotype than *Ghd7* ^Hap2^ in the *indica* population (**Figures 3D-F**).

*DTH7*/*Ghd7.1*/*OsPRR37* encodes a pseudo-response regulator, a major genetic locus affecting the heading date and grain number per panicle (Liu et al., 2013; Koo et al., 2013; Yan et al., 2013; Gao et al., 2014). Based on three non-synonymous SNPs and four promoter SNPs, we detected five haplotypes of *DTH7* in *qGNP7.8*. *DTH7* ^Hap4^ showed higher grain numbers in both *indica* and *japonica* subpopulations, whereas, as inferior haplotypes, *DTH7* ^Hap2^ and *DTH7* ^Hap5^ had fewer grains in the *indica* and *japonica* subpopulations (**Supplementary Figures 5B, C**).

### Candidate gene analysis in *qGNP1.3*

**Figure 4:**
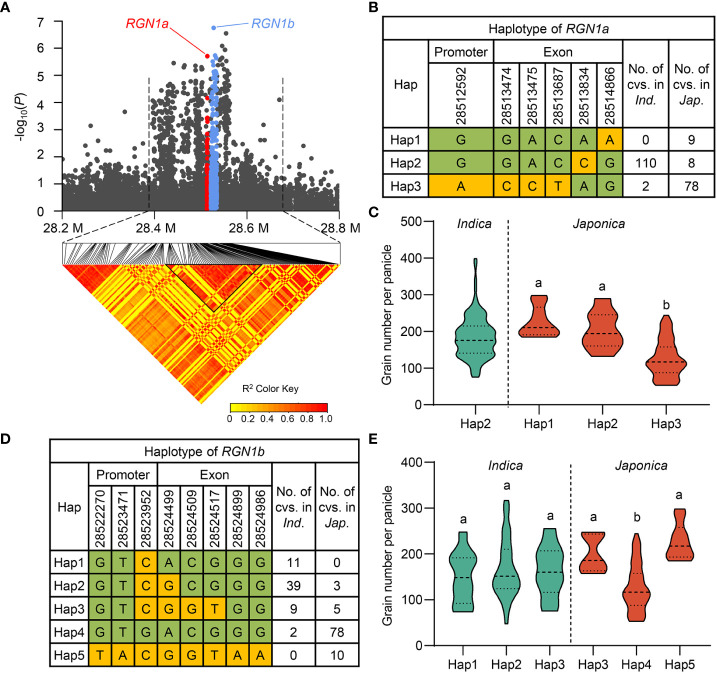
Exploration of *RGN1a* and *RGN1b* for grain number per panicle on chromosome 1. **(A)** Regional Manhattan plot (top) and pairwise LD analysis (bottom) of *qGNP1.3* for GNP on chromosome 1. Red and cornflower blue dots represent all SNPs within *RGN1a* and *RGN1b*, respectively. **(B, D)** Different haplotypes of *RGN1a* or *RGN1b* in the *indica* and *japonica* subgroups. **(C, E)** A comparison of GNP traits among haplotypes of *RGN1a* and *RGN1b* in the *indica* and *japonica* subgroup. In **(C, E)**, the green violins represent *indica*, the red violins represent *japonica* rice, and different letters indicate significant differences (*P* < 0.05) detected by one-way ANOVA.

As shown above, three cloned genes regulating grain number were detected in our GWAS results for the full population. Given that *qGNP1.1* containing *TUT1* and *qGNP7.1* containing *Ghd7* were both identified in at least two years, we focused on QTLs that were detected multiple times in the full population (**Figure 2**; **Table 1**). *qGNP1.3* showed the strongest signal in GWAS (**Figure 2**) and has been identified as having a major effect on grain number and secondary branches using linkage mapping (Deshmukh et al., 2010; Zhu et al., 2013). Candidate genes in a 120 kb region were screened, and five genes with non-synonymous SNPs (-log(*P*) ≥ 3) were identified (**Supplementary Figure 6**). Genes regulating grain number per panicle must be expressed during the young panicle development period. We compared the expression levels of the five genes mentioned using public expression data (Sato et al., 2011; Li et al., 2016), and found that only *LOC_Os01g49580*, *LOC_Os01g49614*, and *LOC_Os01g49680* were expressed in the young panicle (**Supplementary Figure 6**). *qGNP1.3* has been identified by comparing single-segment substitution lines (Nipponbare/NIP introgression segments in Guangluai 4 background) with the recurrent parent Guangluai 4 (Zhu et al., 2013). The significant non-synonymous SNPs of *LOC_Os01g49580* and *LOC_Os01g49614* exhibited polymorphism differences between NIP and Guangluai 4, in addition to *LOC_Os01g49680*. Protein functional analysis revealed that *LOC_Os01g49580* and *LOC_Os01g49614* were both annotated as protein kinase domain-containing proteins, and *LOC_Os01g49680* encodes the DNA repair helicase XPB2 (**Supplementary Figure 6**). Several genes that regulate panicle development in rice encode protein kinases. For example, mitogen-activated protein kinase GSN1, OsMKKK10, OsMKK4, OsMPK6, and the receptor-like protein kinase OsER1 control spikelet number *via* the same pathway (Guo et al., 2018; Guo et al., 2020a), and glycogen synthase kinase 2/GSK2 and glycogen synthase kinase 3/GSK3 participate in phosphorylating or dephosphorylating processes to modulate grain size in rice (Gao et al., 2019; Lyu et al., 2020; Liu et al., 2021). These results prompted us to select *LOC_Os01g49580* and *LOC_Os01g49614* as candidate genes, named *RGN1a* and *RGN1b*, respectively (**Figure 4A**). Haplotype analysis of *RGN1a* or *RGN1b* showed they both had significant genetic variation in GNP in *japonica* subgroups (**Figures 4B–E**).

### *RGN1a* regulates the grain number per panicle in rice

Since *RGN1a* and *RGN1b* both belong to the protein kinase family, we first conducted amino acid sequence alignments between them. Both had a wall-associated receptor kinase N-terminal domain and a catalytic domain of the serine/threonine kinases and shared 95.43% similarity with the C-terminal structure (RGN1a, aa 562-913; RGN1b, aa 316-666) (**Supplementary Figure 7**). Further protein sequence alignments of RGN1a, RGN1b, and their homologs revealed that they are highly conserved in monocots (**Supplementary Figure 8**). This indicates that these two genes may perform similar functions in rice.

**Figure 5:**
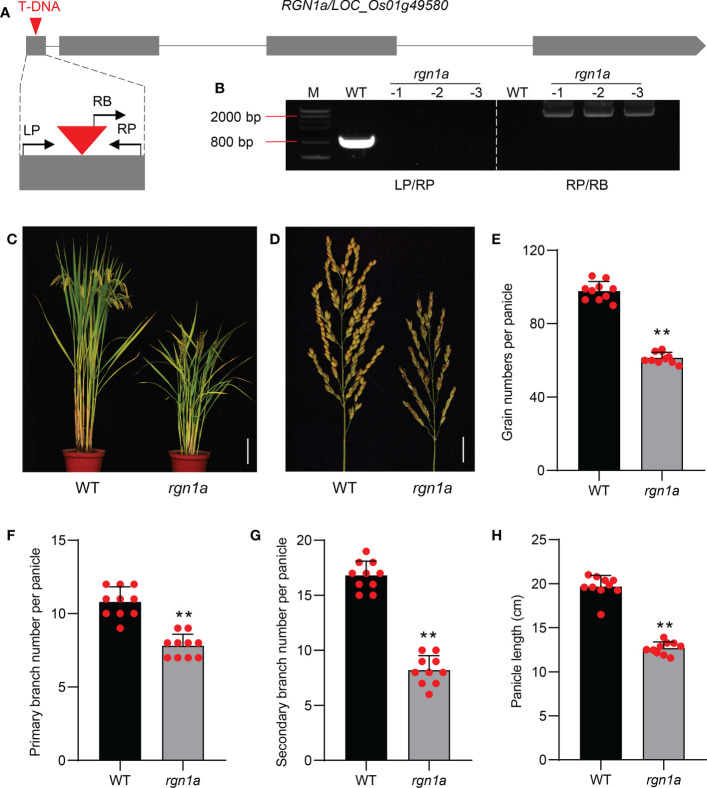
Identification and characterization of *RGN1a* controlling panicle development. **(A)** Schematic diagram of *RGN1a* gene with T-DNA insertion. Gray boxes and lines represent exons and introns, respectively. The insertion sites were in the first exon of *RGN1a*. LP and RP are the primers in the gene body, and RB is the primer in the T-DNA element. **(B)** PCR-analysis of WT and three *rgn1a* mutants using LP/RP and RB/RP. **(C)** Comparison of plant architecture of the wild type and *rgn1a*. Scale bar = 15 cm. **(D)** Comparison of panicle architecture of wild type and *rgn1a*. Scale bar = 2 cm. **(E–H)** Comparison of grain number per panicle, primary branch number per panicle, secondary branch number per panicle, and panicle length between wild and *rgn1a* mutant. *P*-values were determined using two-tailed Student’s *t*-tests. **P < 0.01. The data are shown as mean ± SD (*n* = 10).

To validate the function of *qGNP1.3* in regulating grain number, we first obtained a T-DNA insertion mutant *rgn1a* in the Dongjin/DJ background. The T-DNA mutant *rgn1a* was accurately identified by electrophoresis and sequencing, showing that the T-DNA element was inserted in the first exon of *RGN1a* (**Figures 5A, B**). The grain number per panicle, primary branch, secondary branch per panicle, and panicle length of *rgn1a* significantly decreased by 37.2%, 27.8%, 51.2%, and 25.5%, respectively, compared to the wild type (**Figures 5C–H**). Comparison of GNP of individuals acquired by selfing from heterozygous T-DNA insertion mutant containing *RGN1a*, *RGN1a*/*rgn1a*, and *rgn1a* alleles, indicating that T-DNA insertion in *RGN1a* was co-segregated with the phenotype of grain number (**Supplementary Figure 9**). We also tested the expression levels of *RGN1a* in germplasm materials containing Hap2 and Hap3, and found no significant differences (**Supplementary Figure 10**). These results indicate that the natural functional variations of *RGN1a* are located in the coding region.

### Breeding application of *RGN1a* and *DTH7* in the *japonica* subpopulation

**Figure 6:**
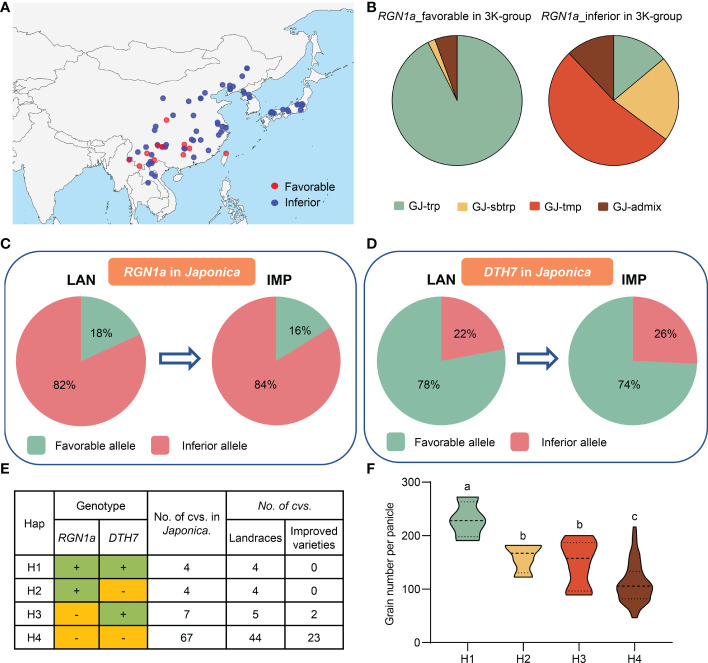
Breeding utilization of *RGN1a* and *DTH7* in grain number per panicle. **(A)** The geographical distribution of *RGN1a* alleles among *japonica* varieties in Asia. **(B)** The frequency of *RGN1a* alleles among four *japonica* subgroups. **(C)** Allelic changes in *RGN1a* during *japonica* rice breeding. **(D)** Allelic changes in *DTH7* during *japonica* rice breeding. **(E)** Combined haplotype analysis of *RGN1a*-*DTH7* in the *japonica* subpopulation, ‘+’ and ‘–’ indicate favorable and inferior alleles. **(F)** Comparison of GNP trait among combined haplotypes of *RGN1a* and *DTH7* in *japonica* subgroup. Different letters indicate significant differences (*P* < 0.05) detected by one-way ANOVA.

We detected three haplotypes at *RGN1a*, but it only had *japonica*-specific allele variations for grain number per panicle, and no haplotype differences were present in *indica* accessions (**Figures 4B, C**). The favorable haplotype of *RGN1a* (Hap1 and Hap2) in *japonica* accessions contained 17 varieties, whereas the inferior haplotype of *RGN1a* (Hap3) contained 78 *japonica* germplasm resources (**Figure 4B**). We analyzed the geographical distribution of these materials in Asia and found varieties with favorable alleles that were mainly distributed in southwest China (**Figure 6A**). This result indicates that the different genotypes of *RGN1a* show regional distribution specificity. Furthermore, using information from four *japonica* clusters (temperate *japonica*/GJ-tmp, subtropical *japonica*/GJ-sbtrp, tropical *japonica*/GJ-trp, and admix *japonica*/GJ-admix) in the 3K-RG accessions (Wang et al., 2018), we analyzed the ascription of different genotypes of *RGN1a*. The varieties containing inferior *RGN1a* were present in all four *japonica* subpopulations. However, the varieties containing favorable alleles of *RGN1a* mainly belonged to the subtropical and tropical *japonica* subgroups (**Figure 6B**). These results implied that favorable alleles of *RGN1a* were more suitable for the growing conditions in subtropical and tropical regions and had not been used in temperate regions.

In this study, three cloned genes, *TUT1*, *Ghd7*, and *DTH7*, were significantly associated with GNP, and we identified a new gene, *RGN1a*, which also controlled panicle development. Both *RGN1a* and *DTH7* showed considerable genetic variation in the *japonica* subpopulation. The favorable alleles of *RGN1a* and *DTH7* had opposite proportions in landraces (18% and 84%, respectively) and improved varieties (16% and 74%, respectively). This indicated that *DTH7* had been widely used to improve grain number, and the utilization of *RGN1a* remains undeveloped (**Figures 6C, D**; **Supplementary Figure 11A**). We also conducted a joint haplotype analysis of *RGN1a* and *DTH7*. A panel of 82 *japonica* accessions was divided into four haplotypes. H1 contained the favorable alleles *RGN1a* and *DTH7* and showed the highest grain numbers. H2 with favorable *RGN1a* and H3 with favorable *DTH7* exhibited similar phenotypes but less grain numbers than H1. H4, with inferior *RGN1a* and *DTH7*, had the lowest grain numbers (**Figures 6E, F**). This result confirmed that variety H1 containing favorable alleles of *RGN1a* and *DTH7* could effectively increase the grain number per panicle. However, varieties of H1 are all present in landraces, indicating a broad prospect for the aggregation and utilization of *RGN1a* and *DTH7* in improved species.

## Discussion

### *RGN1a* is a novel gene that regulates the grain number per panicle

Grain yield is a vital research direction that needs continuous attention; however, as a complex quantitative trait, it is difficult to excavate eximious alleles in a natural population. GWAS is considered an effective method for gene mining. In recent years, many genes that control grain yield have been cloned into rice. *OsSPL13*, a transcription factor that positively regulates grain length, was identified in a GWAS performed on a *japonica* population containing 381 varieties (Si et al., 2016). *GSE5*, participating in regulating cell proliferation in spikelet hulls and controlling the grain size, was identified using a GWAS approach (Duan et al., 2017). Because the grain number per panicle is determined by panicle length, primary branch, and secondary branch, any changes in them will affect the GNP. To date, few GNP genes have been mapped through GWAS.

In this study, based on phenotypic and high-quality sequencing data, we performed GWAS in the full rice population and found a target gene, *RGN1a*, encoding a protein containing the catalytic domain of the serine/threonine kinases and wall-associated receptor kinase N-terminal domain. *qGNP_J_1.3*, containing *RGN1a*, was identified in the GWAS results of the *japonica* subgroup (**Supplementary Table 2**), indicating that the genetic variation in *RGN1a* was mainly concentrated in the *japonica* subgroup. A T-DNA insertion mutant of *RGN1a* in Dongjin (a *japonica* variety) background affected both panicle branch and grain numbers (**Figures 5D–H**), suggesting that *RGN1a* is a novel gene involved in the regulation of GNP. In addition, we also found RGN1a participates in regulating plant height and seed setting rate through phenotype investigation.

### RGN1a can be classified as a novel OsWAK-RLCK protein

The cell wall-associated kinase (WAK) family plays an important role in cell expansion and disease resistance (Lally et al., 2001; Verica and He, 2002; Li et al., 2009; Zuo et al., 2015; Delteil et al., 2016) and is mainly composed of extracellular domains (wall-associated receptor kinase galacturonan-binding (GUS-WAK-bind) domain and epidermal growth factor (EGF)), cytoplasmic Ser/Thr kinase domain, and a transmembrane region. The conserved WAK proteins, maintaining approximately 80% similarity, are mainly expressed in the cytoplasmic kinase domain. In contrast, the sequences of five WAK genes in the extracellular domains of *Arabidopsis* shared only 40% to 64% identity (He et al., 1999; Zhang et al., 2005).

A total of 125 WAKs were identified from the rice genome of *japonica* Nipponbare and divided into five gene types: OsWAK-RLK, OsWAK-RLCK, OsWAK-RLP, OsWAK short gene, and OsWAK pseudogene (Zhang et al., 2005). Meanwhile, OsWAK-RLCK was defined as having only a cytoplasmic protein kinase domain with more than 40% identity to an OsWAK-RLK member. RNAi-mediated silencing of *OsiWAK1* results in decreased plant height, pollen fertility, and flowers per panicle (Kanneganti and Gupta, 2011). Silencing *DEFECT in RARLY EMBRYO SAC1* (*OsDEES1*) causes a functional defect in early embryo sac development and reduced pollen fertility (Wang et al., 2012). *OsWAK11* influences grain size and leaf angle by regulating cell elongation rate (Yue et al., 2022). In our study, RGN1a lacked the extracellular domain of EGF compared to WAKs. While the T-DNA mutant *rgn1a* showed a phenotype similar to *that of the OsiWAK1* or *OsDEES1* RNAi lines, the grain number per panicle greatly decreased compared to the wild type (**Figures 5C-H**). Therefore, we believe that RGN1a can be classified as a novel OsWAK-RLCK protein, although further molecular experiments are needed to verify its kinase activity.

### *RGN1a* and *RGN1b* gene clusters might be utilized in *japonica* breeding in China

Based on candidate gene analyses in *qGNP1.3*, the amino acid sequence of *RGN1b* was found to be highly homologous to *RGN1a* (**Supplementary Figure 7**). Both genes have a GUS-WAK-binding domain and a cytoplasmic kinase domain and are distributed within a 23 kb region on chromosome 1. Interestingly, WAK genes and WAK-like genes in *Arabidopsis* usually lie in a tight cluster, such as *WAK1*-*WAK5*, *WAKL1*-*WAKL8*, and *WAKL11*-*WAKL13*, located in a region spanning less than 12 cM (He et al., 1999; Verica and He, 2002);. Gene structure and expression analyses of the rice WAK gene family revealed that localized gene duplication resulted in expanded rice (Zhang et al., 2005). Our results showed that *RGN1a* and *RGN1b* are closely distributed on chromosome 1, providing new evidence for this conclusion.

Comparing the geographical distribution of rice germplasm in the favorable haplotype of *RGN1a* or *RGN1b*, these varieties are mostly located in the Yunnan, Guizhou, Hunan, Shanxi, and Guangxi provinces of China (**Supplementary Figures 11A, B**). *RGN1a* and *RGN1b* are adjacent to a ~23 kb block (**Supplementary Figure 12**), indicating that *RGN1a* and *RGN1b* were linked and received common selection in evolutionary history. Moreover, *rgn1a* reduced grain number, indicating that *RGN1b* may also regulate GNP. In the future, we also need to construct *rgn1b* and double *RGN1a* and *RGN1b* mutants to explore whether there is an additive effect on GNP regulation of these two genes.

We also discussed the breeding applications of *RGN1a* and *RGN1b* for variety improvement. The favorable alleles of *RGN1a* and *RGN1b* occupied a large proportion of *indica* and suggested that they had been widely used in the *indica* subgroup (**Figures 4B–E**). However, the favorable alleles of *RGN1a* and *RGN1b* in the *japonica* group were mostly found in landraces, especially in China (**Supplementary Figures 11A, B**). The few varieties containing favorable alleles of *RGN1a* or *RGN1b* located abroad were mainly improved varieties (**Supplementary Figure 11**). This result encourages us to use *RGN1a* and *RGN1b* in the genetic improvement of the grain number per panicle of *japonica* rice in China.

## Conclusions

Here, we performed a GWAS for the grain number per panicle in the full rice population using a compressed mixed linear model. In our results, *Ghd7*, *DTH7*, and *TUT1* were significantly associated with GNP. A novel region*, qGNP1.3*, contributes to the genetic variation of GNP in the *japonica* subgroup. The transgenic phenotype confirmed that *RGN1a* positively regulated the grain number per panicle. Favorable alleles of *RGN1a* have been used in the *indica* population, and the aggregation of *RGN1a* and *DTH7* in the *japonica* population showed additive effects on GNP. There is no doubt that there will be a large increase in grain number per panicle when favorable *RGN1a* and other reported genes are utilized in improved *japonica* varieties.

## Data availability statement

The raw data supporting the conclusions of this article will be made available by the authors, without undue reservation.

## Author contributions

QZ and HZ designed the research. QZ performed most of experiments, and wrote the manuscript. QZ, JX, and XW performed data analysis. XW and XZ investigated the phenotype. ML and TY performed part of the experiments. CS, JL, and ZL provided technical assistance. NK assisted with revisions for the manuscript. ZZ and HZ provided funding support and supervised the manuscript. All authors contributed to the article and approved the submitted version.
