# Supplementary material for: Natural variation of RGN1a regulates grain number per panicle in japonica rice
Source: Front Plant Sci. 2022 Dec 13;13:1097622. doi: 10.3389/fpls.2022.1097622 (PMC9795840; doi:10.3389/fpls.2022.1097622)
Supplement: Supplementary file 1 [file DataSheet_1.pdf]

Natural variation of *RGN1a* regulates grain number per panicle in *japonica* rice

Quan Zhang[1,†], Jianyin Xie[1,2,†],Xueqiang Wang[1,†], Miaosong Liu[1], Xiaoyang Zhu[1], Tao Yang[1], Najeeb Ullah Khan[1], Chen Sun[1], Jinjie Li[1], Zhanying Zhang[1], Zichao Li[1,3], Hongliang Zhang[1,2,3*]

[1] State Key Laboratory of Agrobiotechnology/Beijing Key Laboratory of Crop Genetic Improvement, China Agricultural University, Beijing, China.

[2] Sanya Institute of China Agricultural University, Sanya Nanfan Research Institute of Hainan University, Sanya, China.

[†]. These authors contributed equally to this work

[*]. Corresponding author: zhangl@cau.edu.cn

Supplementary material

Supplementary Figure 1. Kinship analyses of 266 rice accessions

Supplementary Figure 2. The distribution of grain number per panicle in *indica* and *japonica* subpopulations among different years

Supplementary Figure 3. GWAS results of GNP in *indica* and *japonica* subpopulations among different years

Supplementary Figure 4. The significant loci distribution among different GWAS populations

Supplementary Figure 5. Exploration of *DTH7* for grain number per panicle

Supplementary Figure 6. Details for five potential candidate genes in *qGNP1.3*

Supplementary Figure 7. The amino acid alignments among RGN1a, RGN1b, and other homologous proteins from monocots

Supplementary Figure 8. Phylogenetic analysis of RGN1a protein in monocots

Supplementary Figure 9. Comparison of GNP among segregating population containing *RGN1a*, *RGN1a*/*rgn1a*, and *rgn1a* allele

Supplementary Figure 10. The comparison of expression level of *RGN1a* between Hap2 and Hap3.

Supplementary Figure 11. The geographical distribution of favorable *RGN1a* and *RGN1b*

Supplementary Figure 12. The Linkage disequilibrium analysis between *RGN1a* and *RGN1b*

Supplementary Table 1. Primers used in the study

Supplementary Table 2. Description of 16 QTLs for GNP in *indica*, *japonica* subpopulations of 2010_HN, 2012_HN, and 2013_HN

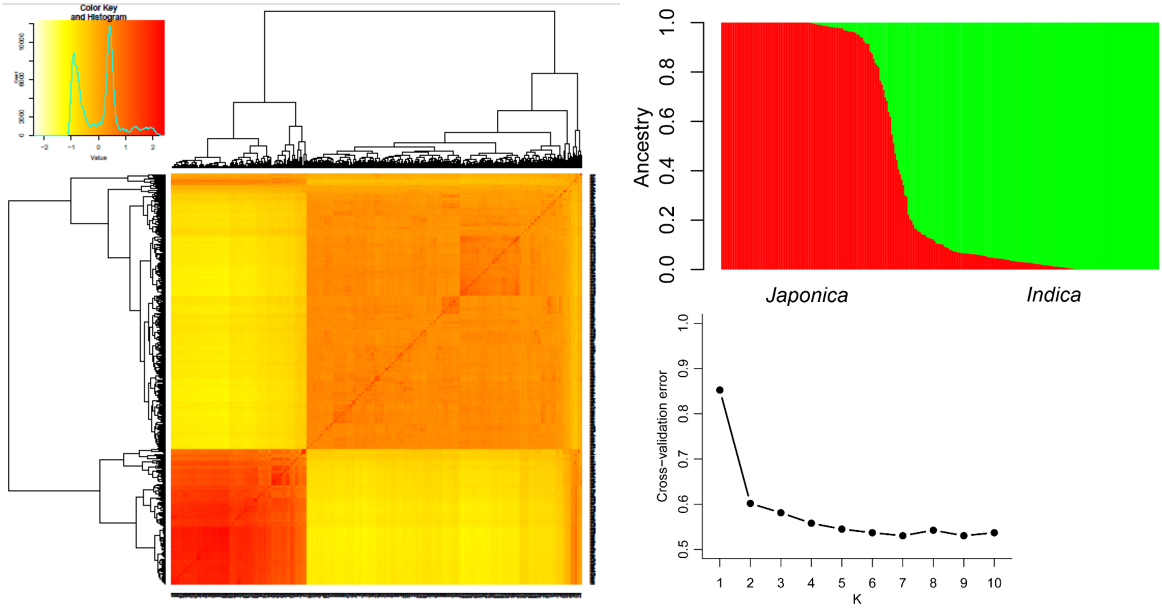

Supplementary Figure 1. Kinship analyses of 266 rice accessions.

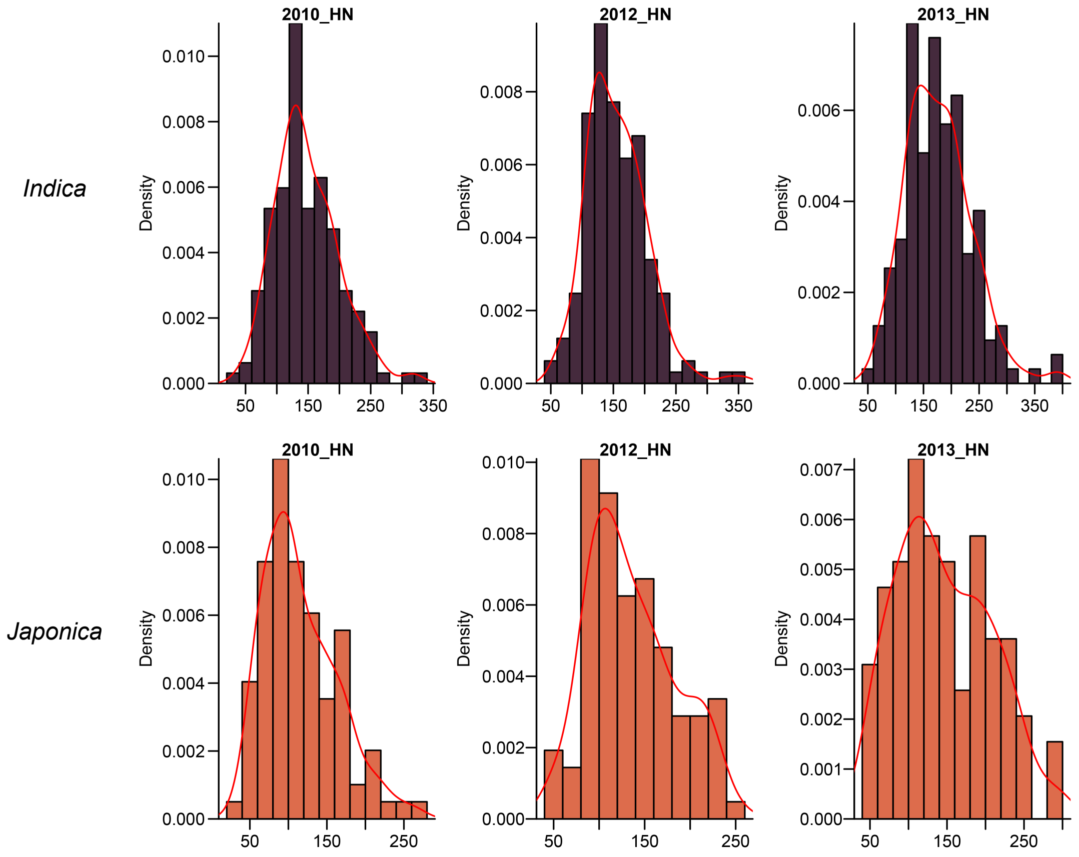

Supplementary Figure 2. The distribution of grain number per panicle in *indica* and *japonica* subpopulations among different years

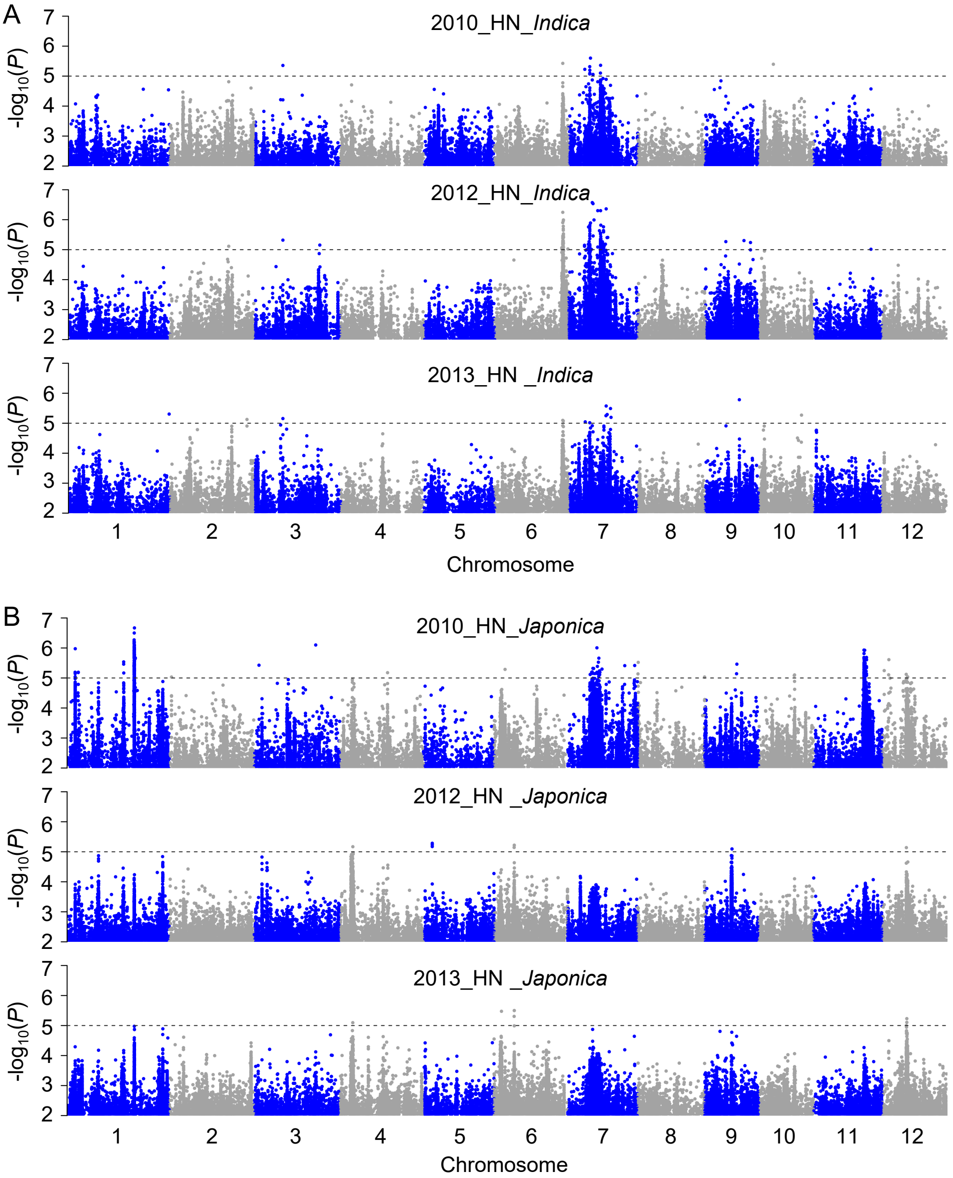

Supplementary Figure 3. GWAS results of GNP in *indica* and *japonica* subpopulations among different years. (A) Manhattan plots for the GWAS in indica subpopulations among different years. **(B)** Manhattan plots for the GWAS in *japonica* subpopulations among different years. Dashed horizontal line for each figure indicates the significance threshold ($P = 10^{-5}$).

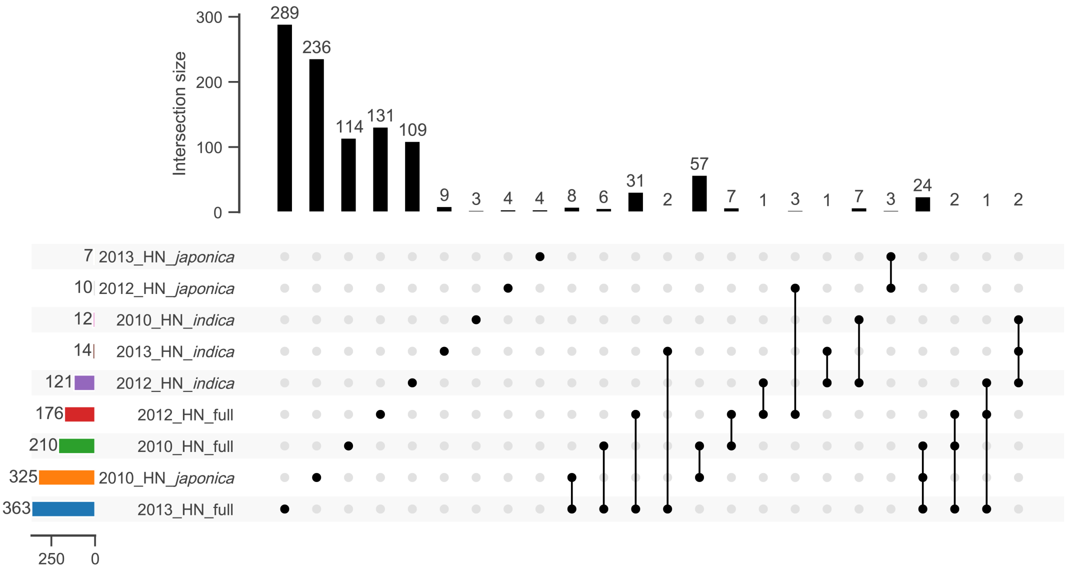

Supplementary Figure 4. The significant loci distribution among different GWAS populations. The left 9 columns of picture showed the number of unique loci in every GWAS populations, the right 15 columns of picture showed the common loci among GWAS populations.

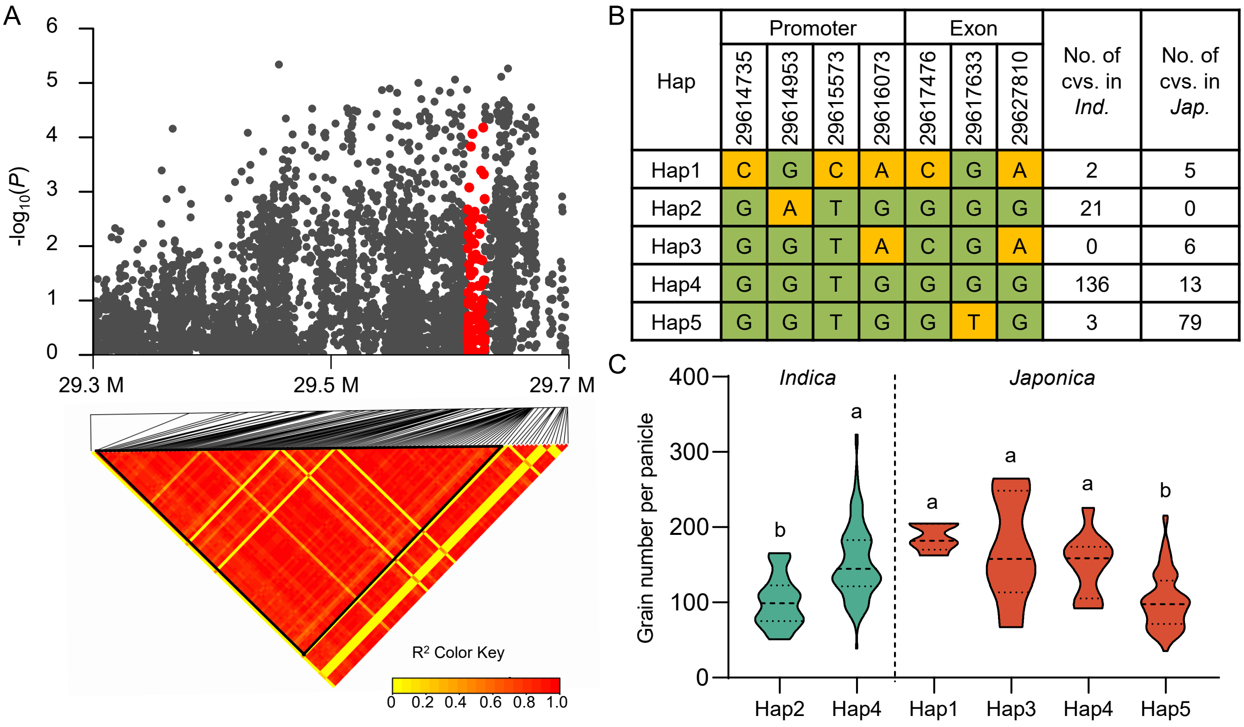

Hap	Promoter				Exon			No. of cvs. in *Ind.*	No. of cvs. in *Jap.*
	29614735	29614953	29615573	29616073	29617476	29617633	29627810		
Hap1	C	G	C	A	C	G	A	2	5
Hap2	G	A	T	G	G	G	G	21	0
Hap3	G	G	T	A	C	G	A	0	6
Hap4	G	G	T	G	G	G	G	136	13
Hap5	G	G	T	G	G	T	G	3	79

Supplementary Figure 5. Exploration of *DTH7* for grain number per panicle. (**A**) Regional Manhattan plot (top) and pairwise LD analysis (bottom) of *qGNP7.8* containing *DTH7* for GNP. Red dots represent all SNPs within *DTH7*. (**B**) Different haplotypes of *DTH7* in *indica* and *japonica* subgroup. (**C**) Comparison of GNP trait among haplotypes of *DTH7* in *indica* and *japonica.* In (**C**), the green violins represent *indica* and red violins represent *japonica* rice, and different letters indicate significant differences ($P < 0.05$) detected by one-way ANOVA.

Gene_ID	The prediction of protein function	Gene expesssion in panicle			Sig_non_sys between Guangluai 4 and Nipponbare
		93-11	PA64S	RiceXpro	
LOC_Os01g49580	Protein kinase domain containing protein, expressed	√	√	√	√
LOC_Os01g49614	Protein kinase domain containing protein, expressed	√	√	√	√
LOC_Os01g49630	proteophosphoglycan ppg4, putative, expressed	/	/	/	/
LOC_Os01g49660	reticulon domain containing protein, putative, expressed	/	/	/	/
LOC_Os01g49680	DNA repair helicase XPB2, putative, expressed	√	√	√	/

Supplementary Figure 6. Details for five potential candidate genes in *qGNP1.3*

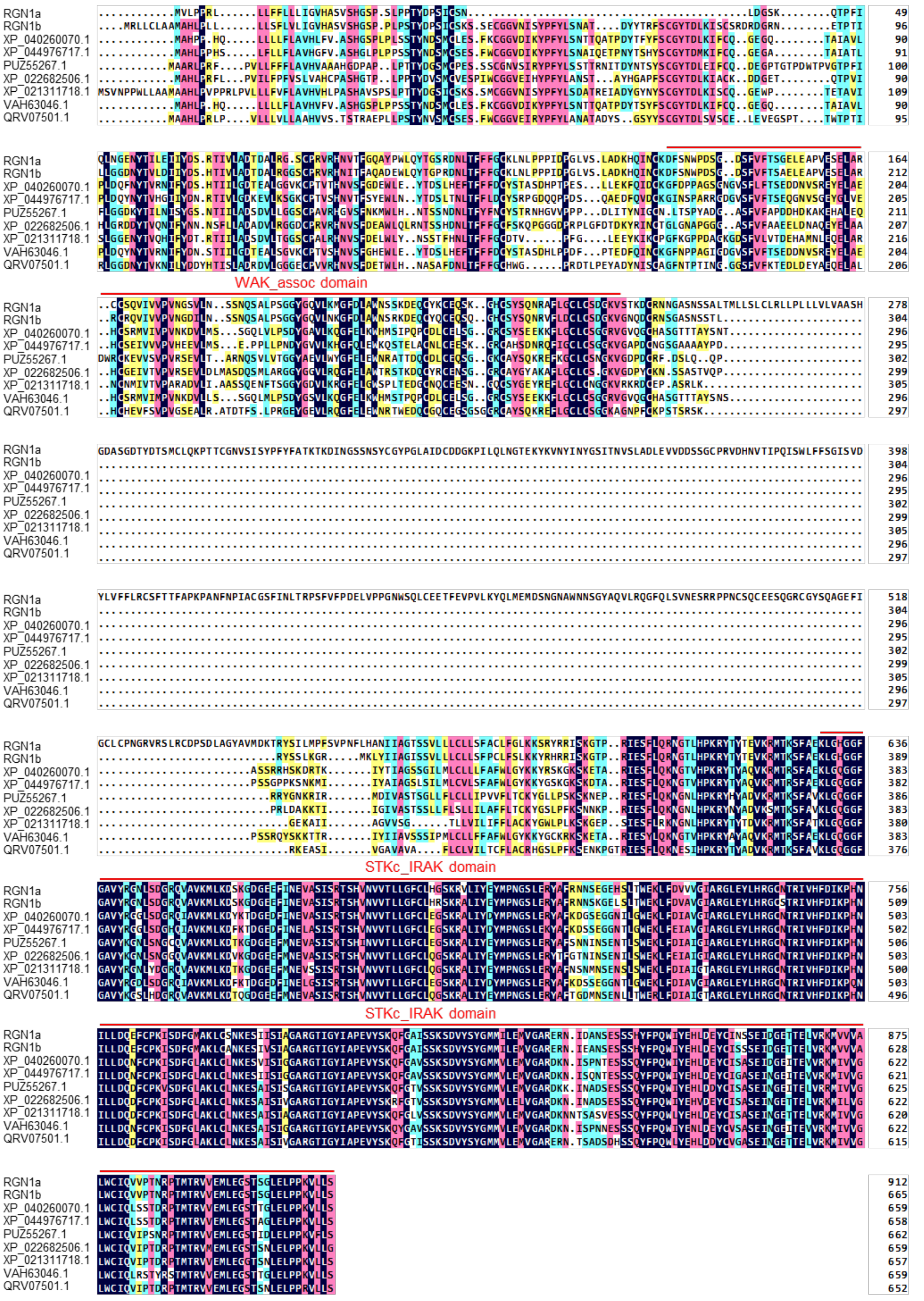

Supplementary Figure 7. The amino acid alignments among RGN1a, RGN1b, and other homologous proteins from monocots. The protein sequences were downloaded from NCBI (https://www.ncbi.nlm.nih.gov/).

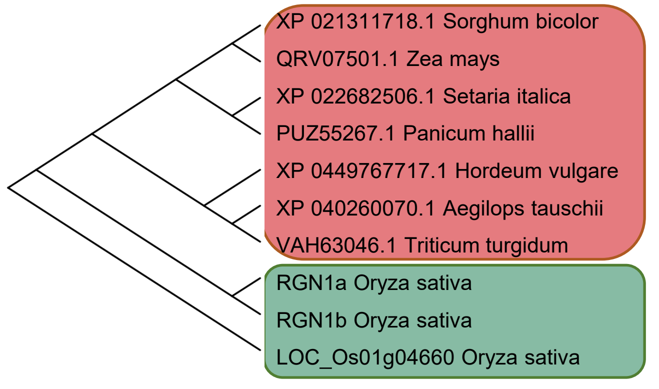

Supplementary Figure 8. Phylogenetic analysis of RGN1a protein in monocots.

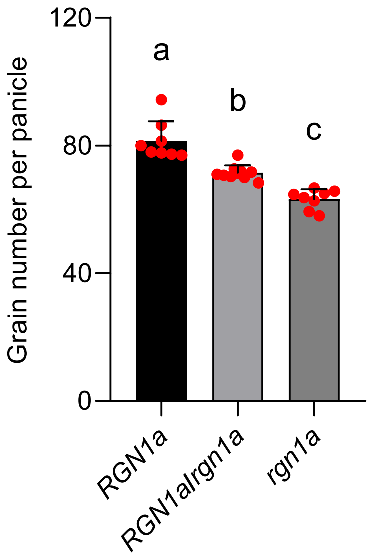

Supplementary Figure 9. Comparison of GNP among segregating population containing *RGN1a*, *RGN1a*/*rgn1a*, and *rgn1a* allele. Different letters indicate significant differences (P < 0.05) detected by one-way ANOVA.

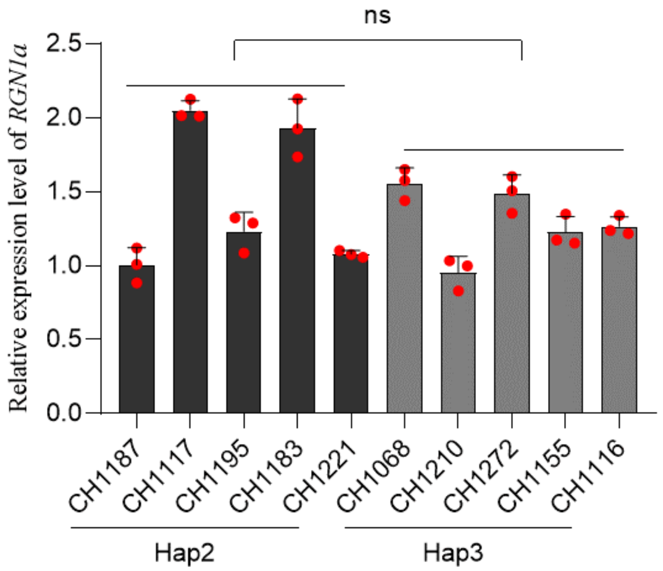

Supplementary Figure 10. The comparison of expression level of *RGN1a* between Hap2 and Hap3. Expression level of *RGN1a* in young panicle among germplasm materials. Statistical analysis were determined using Student’s *t*-test.

A

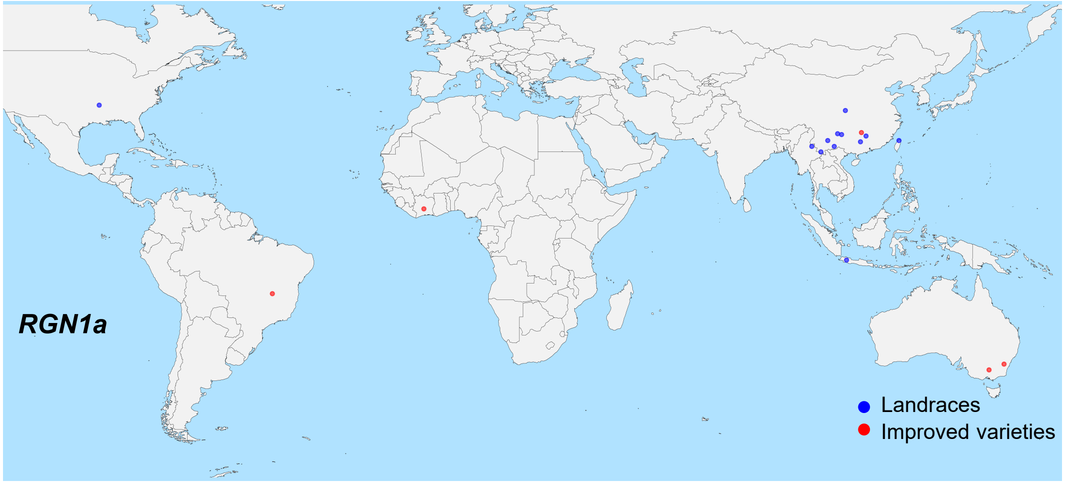

B

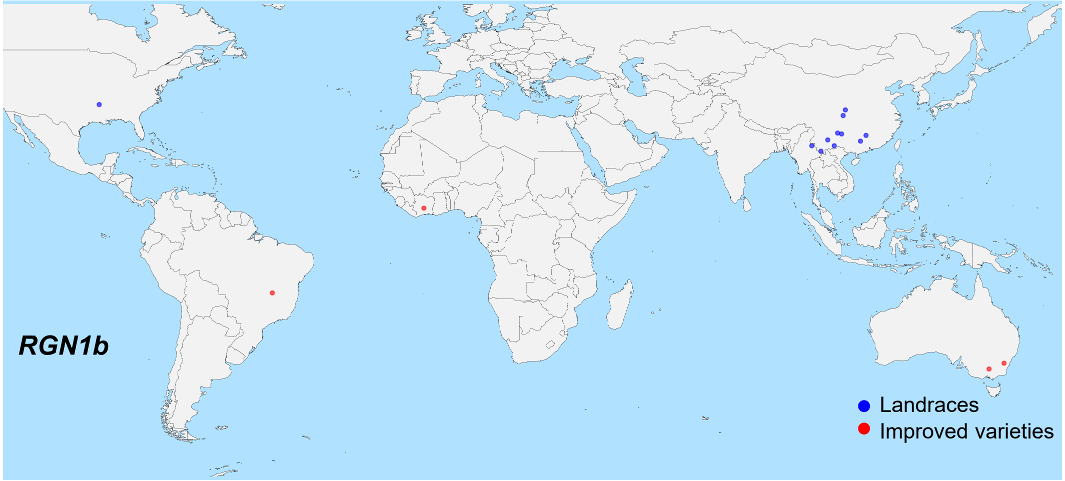

Supplementary Figure 11. The geographical distribution of favorable *RGN1a* and *RGN1b*. (A) The geographical distribution of favorable *RGN1a*. **(B)** The geographical distribution of favorable *RGN1b*. The blue and red dots present landraces and improved varieties, respectively.

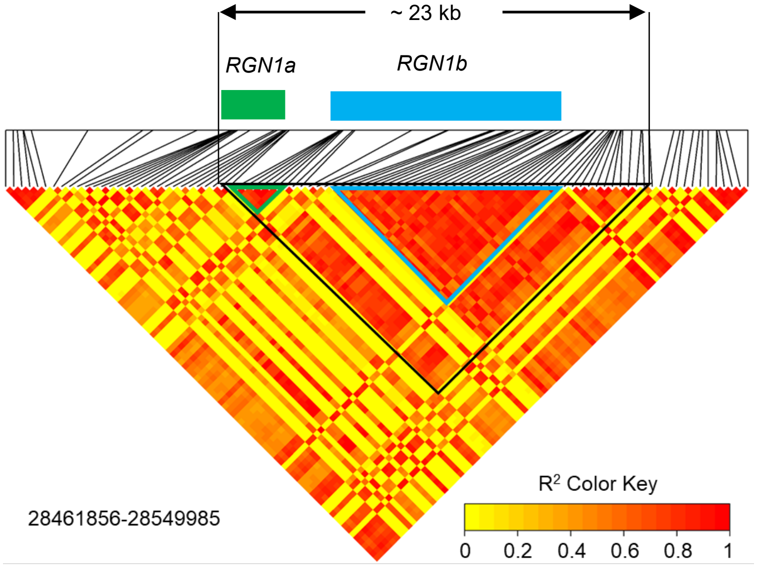

Supplementary Figure 12. The Linkage disequilibrium analysis between *RGN1a* and *RGN1b*. The green and blue triangles represent *RGN1a* and *RGN1b*, respectively. The black triangle shows that *RGN1a* and *RGN1b* belong to the same block.

Supplementary Table 1. Primers used in the study

Name	sequence
LP	AAGCGAACAGAAACACGG
RB	CAAAGCCAGTAAAGTAGAACGG
RP	CGATGACTTGATGCTTTGTG
RGN1a-RT-F	GTGCTGTTTACAGAGGCAACCTAT
RGN1a-RT-R	CCGTCACCCTTGGAGTCCTTT

Supplementary Figure 2. Description of 16 QTLs for GNP in *indica*, *japonica* subpopulations of 2010_HN, 2012_HN, and 2013_HN.

QTL	Env	Chr	Left_Position	Right_Position	Leader_SNP	*P*_value	Cloned Gene
qGNP_I_6.1	2012_HN_*Indica*	chr6	28494361	28617332	28494361	8.58E-06	
qGNP_I_6.2	2012_HN_*Indica*	chr6	28895574	29224895	28925635	5.67E-07	
qGNP_I_7.1	2012_HN_*Indica*	chr7	8543895	8619143	8617530	1.74E-06	
qGNP_I_7.2	2012_HN_*Indica*	chr7	8836538	8937289	8857359	5.37E-06	
qGNP_I_7.3	2010_HN_*Indica*	chr7	9138804	9158833	9138804	4.82E-06	*Ghd7*
	2012_HN_*Indica*	chr7	9138804	9193051	9157017	1.51E-06	
qGNP_I_7.4	2012_HN_*Indica*	chr7	13617052	13897269	13723347	4.95E-07	
qGNP_I_7.5	2012_HN_*Indica*	chr7	14492934	14748935	14500081	5.42E-06	
qGNP_I_7.6	2012_HN_*Indica*	chr7	14989964	15057390	14990166	1.72E-06	
qGNP_J_1.1	2010_HN_*Japonica*	chr1	3376152	3445396	3445396	1.05E-06	
qGNP_J_1.2	2010_HN_*Japonica*	chr1	23998771	24008783	23998771	2.90E-06	
qGNP_J_1.3	2010_HN_*Japonica*	chr1	28322981	28562031	28555223	2.13E-07	
qGNP_J_5.1	2012_HN_*Japonica*	chr5	3759829	3774726	3774726	5.21E-06	
qGNP_J_6.1	2012_HN_*Japonica*	chr6	8529373	8596219	8596218	6.03E-06	
	2013_HN_*Japonica*	chr6	8529373	8596219	8596218	3.15E-06	
qGNP_J_7.1	2010_HN_*Japonica*	chr7	13051699	13241516	13241516	2.18E-06	
qGNP_J_7.2	2010_HN_*Japonica*	chr7	13477371	13664377	13477371	5.78E-06	
qGNP_J_11.1	2010_HN_*Japonica*	chr11	21370948	21997086	21392821	1.17E-06	
	2010_HN_*Japonica*	chr11	22292783	22458360	22449936	2.05E-06	
